# Influence of Fiber Angle on Steady-State Response of Laminated Composite Rectangular Plates

**DOI:** 10.3390/ma15165559

**Published:** 2022-08-12

**Authors:** Ahmad Saood, Arshad Hussain Khan, Md. Israr Equbal, Kuldeep K. Saxena, Chander Prakash, Nikolay Ivanovich Vatin, Saurav Dixit

**Affiliations:** 1 Mechanical Engineering Section, Faculty of Engineering & Technology, University Polytechnic, AMU, Aligarh 202002, India; 2 Department of Mechanical Engineering, ZHCET, AMU, Aligarh 202002, India; 3 Department of Mechanical Engineering, GLA University, Mathura 281406, India; 4 School of Mechanical Engineering, Lovely Professional University, Phagwara 144001, India; 5 Division of Research and Development, Lovely Professional University, Phagwara 144001, India; 6 Peter the Great St. Petersburg Polytechnic University, 195251 Saint Petersburg, Russia; 7 Division of Research & Innovation, Uttaranchal University, Dehradun 248007, India

**Keywords:** composite, nonlinear forced vibration, shooting technique, steady-state

## Abstract

Significant advances in the field of composite structures continue to be made on a variety of fronts, including theoretical studies based on advances in structural theory kinematics and computer models of structural elements employing advanced theories and unique formulations. Plate vibration is a persistently interesting subject owing to its wider usage as a structural component in the industry. The current study was carried out using the C^o^ continuous eight-noded quadrilateral shear-flexible element having five nodal degrees of freedom, which is ground on first-order shear deformation theory (FSDT). For small strain and sufficiently large deformation, the geometric nonlinearity is integrated using the Von Kármán assumption. The governing equations in the time domain are solved employing the modified shooting technique along with an arc-length and pseudo-arc-length continuation strategy. This work explored the effect of fiber angle on the steady-state nonlinear forced vibration response. To explain hardening nonlinearity, the strain and stress fluctuation throughout the thickness for a rectangular laminated composite plate is determined. The cyclic fluctuation of the steady-state nonlinear normal stress during a time period at the centre of the top/bottom surfaces is also provided at the forcing frequency ratio of peak amplitude in a nonlinear response. Because of the variation in restoring forces, the frequency spectra for all fiber angle orientations show significantly enhanced harmonic participation in addition to the fundamental harmonic.

## 1. Introduction

Theoretical studies incorporating advancements in the kinematics of structural theories, as well as damage and life prediction models, are among the new developments in the field of composites and structures. Advanced theories, innovative formulations, and experimental studies are used to characterize materials and damage models in computer modelling of structural components. Plate vibration is a persistently interesting topic due to its widespread application as a structural component. A finite element (FE) model for the large amplitude vibration of thin plates under harmonic loads was provided [1]. If the right harmonics are not incorporated, the harmonic balance or incremental harmonic balance approaches provide incorrect results [2]. The modal interaction between two modes in laminated plates exposed to harmonic force was studied by Abe et al. [3]. The geometrically nonlinear periodic response analysis of thin rectangular plates exposed to external harmonic excitations was examined by employing the hierarchical finite element approach and the harmonic balance methods. Internal resonance caused modal coupling, and the resulting multimodal and multifrequency responses were verified [4]. Using a C^1^ eight-noded shear-flexible plate element, the dynamic instability of composite laminated plates placed on elastic foundations and exposed to periodic in-plane stresses was investigated [5]. This feature was based on recent kinematics, which allowed for accurate control of the interfaces between the layers of the laminate for displacements and stresses. Using Galerkin’s method, the differential equations of motion were solved, assuming that the transverse deflection had two linear modes. Ganapathi et al. [6] investigated the flexural loss factors of composite laminated beams using the C^1^ quadrilateral eight-noded shear-flexible plate element. The FEM formulation incorporates the impact of anisotropy and in-plane rotatory inertia. The geometrically nonlinear parametric properties of an isotropic and composite plates were obtained utilising a FE framework. The resultant nonlinear equations of motion were solved employing the Newmark time integration approach in combination with a modified Newton–Raphson iteration method [7]. Makhecha et al. [8] employed a novel higher-order theory representing the actual change of in-plane and transverse displacements via thickness for the dynamic response investigation of thick multilayered laminated plates. Geometrically nonlinear vibrations of the rectangular plates exposed to radial harmonic force in the spectral vicinity of the smallest resonances was analyzed by Amabili [9]. The nonlinear strain displacement relationships of the von Karman were applied. The nonlinear responses are obtained by employing a code, based on the arc-length continuance scheme, which allows the bifurcation analysis. Ganapathi et al. [10] analysed the nonlinear dynamic behaviour of thick composite/sandwich laminates using an accurate higher-order theory. Ribeiro and Duarte [11] presented the response-curve behaviour on changing the fiber angle for laminated composite plates, modelled by the *p*-version FEM. Only periodic oscillations were observed in the absence of in-plane compression.

Direct-time integration techniques need greater processing effort to obtain steady-state solutions for large-scale weakly damped nonlinear systems and are incapable of capturing the unstable branches of the response curves [12]. The periodic solution of the nonlinear system of equations can be obtained by the shooting technique. In contrast to frequency domain approaches, there is no need to assume the harmonics. When compared to direct time-integration approaches, steady-state solution of the equations can be found in hardly three to five iterations. Another characteristic of the shooting approach is the generation of monodromy matrix, which can be used to assess the stability of the response [13]. Thomasa and Bilbao performed geometrically nonlinear vibration analysis of a plate having in-plane boundary conditions [14]. Ibrahim et al. [15] present a method based on shooting methodology and a time-integration strategy to obtain the periodic responses of nonlinear structures directly from the solution of a second-order equation of motion without transforming to first-order.

Amabili [16] investigated laminated composite circular cylindrical shells to assess their nonlinear forced vibration response using higher-order shear deformation theory. The large displacement flexural analysis of the laminated composite skew plates using third-order shear deformation theory (TSDT) and von Karman nonlinearity was examined [17]. Breslavsky et al. [18] employed an approach based on the pseudo-arc-length continuation and collocation scheme to analyze bifurcation and resonance for static deflection as well as free/forced, large-displacement vibrations of a thin rectangular rubber plate under equally distributed load. It is demonstrated that the system of an ordinary differential equation having just quadratic and cubic polynomial components may appropriately explain the behaviour of a rubber plate with both geometrical and material nonlinearities. 

Khan and Patel [19] recently obtained the nonlinear forced vibration response of bimodular rectangular plates and cylindrical panels. Their investigation revealed that the response amplitudes for positive/negative half-cycles differ for bimodular plates and shells. Akhavan and Ribeiro [20] explored the large-amplitude forced vibration of variable stiffness laminated plates with curvilinear fibers. To obtain periodic solutions to the governing equations, a shooting strategy based on the Runge–Kutta fifth-order approach and adaptable step-size control is employed. The Tsai–Wu method was used to predict an onset of the damage. The mechanical behaviour of composite laminated skew plates reinforced with carbon nanotubes subjected to transverse time-dependent load was first investigated by Zhang and Xiao [21]. The plate is made up of multilayer nanocomposites that have been strengthened with single-walled carbon nanotubes (SWCNTs). The Mori–Tanaka approach was used to obtained material properties of carbon nanotube-reinforced composites laminated. Gholami and Ansari [22] examined harmonic excitations of the functionally graded graphene platelet-reinforced composite rectangular plates (FG-GPLRC) with varied edge restrictions. Guo et al. [23] investigated the influence of nonlinear factors on the dynamic behaviour of the laminated composite plates reinforced with graphene. Using Hamilton’s principle and the von Karman distortion theory, the governing equations for a reinforced thin composite graphene plate are developed. To evaluate the nonlinear dynamics of the laminated composite plates reinforced with graphene, bifurcation diagrams, waveform graphs, and phase plane plots have been used. Thakur et al. [24] adapted a computationally efficient C^o^ FE model in combination of the nonpolynomial shear deformation theory (NPSDT) for investigation of forced vibrational behaviour of composite laminated plates.

According to the literature review, analyzing the entire nonlinear steady-state periodic response with stable as well as unstable regimes is computationally difficult and has not been addressed thoroughly. The harmonic balance (HB) or incremental harmonic balance (IHB) methods lead to errors in results in absence of the accurate number of harmonics. In certain applications, where excitation/response amplitudes are larger, linear theories struggle to predict strains/stresses, deflections and frequencies to the optimal degree of precision, and a geometrically nonlinear forced vibration response of the plates is found to predict rich and varied responses not possible with the linear analysis. The shooting technique can be used to generate periodic solutions of the nonlinear systems. In contrast to frequency domain approaches, the number of equations is independent of the harmonics number. Furthermore, steady-state solutions are obtained in much fewer iterations as compared to the direct time-integration approaches. Another advantage of the shooting approach is that a monodromy matrix is generated, which is used for forecasting the solution’s stability. The current work focuses on the implementation of the efficient numerical scheme for geometrically nonlinear forced vibration analysis of composite laminated rectangular plates. Our goal in this study is to investigate the effects of fiber orientation on the dynamic response of rectangular composite laminated plates subjected to periodic excitations.

## 2. Formulation

**Figure 1:**
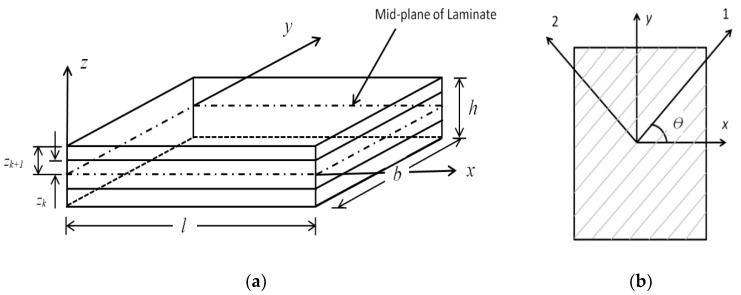
(**a**) Geometric coordinates of the laminated plate; (**b**) Fiber direction for the kth Lamina.

The nonlinear steady-state periodic response analysis of the composite laminated rectangular plates shown in Figure 1a, subjected to a uniformly distributed transverse harmonic force ($F=F_{0}cos\omega_{F}t$) has been studied. In the spectral neighbourhood of the fundamental free vibration frequency, the forcing frequency ($\omega_{F}$) is varied. Using the formulation presented below, the steady-state peak displacement at centre of the plate corresponding to variation in the forcing frequency ratio considering geometrically linear (L) and geometrically nonlinear (NL) strain–displacement relations was obtained.

Using first-order shear deformation theory (FSDT), the displacement field is expressed as:

(1) $$u\left(x,y,z,t\right)=u_{0}\left(x,y,z,t\right)+z\phi_{x}\left(x,y,t\right)\;v\left(x,y,z,t\right)=v_{0}\left(x,y,z,t\right)+z\phi_{y}\left(x,y,t\right)\;w\left(x,y,z,t\right)=w_{0}\left(x,y,t\right)$$

where (*u*_0_, *v*_0_, *w*_0_) are generalized point displacements on the mid-plane; and $\phi_{x}$ and $\phi_{y}$ are rotations of the normal to the mid-plane about the *y* and *x* axes, respectively.

Based on von Karman’s assumption, the strain field in terms of the reference plane may be represented as follows [19]:

(2) $$\left\{\varepsilon \right\}=\left\{\begin{array}{c} \varepsilon_{P}^{L}\\ 0 \end{array}\right\}+\left\{\begin{array}{c} z\varepsilon_{b}\\ \varepsilon_{s} \end{array}\right\}+\left\{\begin{array}{c} \varepsilon_{P}^{NL}\\ 0 \end{array}\right\}$$

where, $\varepsilon_{P}^{L}$ denotes the linear membrane strain, $\varepsilon_{b}$ denotes bending strain, $\varepsilon_{s}$ denotes the transverse-shear strains and $\varepsilon_{P}^{NL}$ denotes the nonlinear membrane-strain tensors, which are further written as [19]:

(3) $$\varepsilon_{p}^{L}=\left\{\begin{array}{c} u_{0,x}\\ v_{0,y}\\ u_{0,y}+v_{0,x} \end{array}\right\};\;\varepsilon_{b}=\left\{\begin{array}{c} \phi_{x,x}\\ \phi_{y,y}\\ \phi_{x,y}+\phi_{y,x} \end{array}\right\};\;\varepsilon_{s}=\left\{\begin{array}{c} \phi_{x}+w_{0,x}\\ \phi_{y}+w_{0,y} \end{array}\right\};$$

The kinetic energy [T(U)] is written in Equation (4), where *ρ_k_* stands for mass density of a *k*th layer and *h_k_*, *h_k +_*_1_ represents thickness parameters of a laminated plate referring to the bottom and top surfaces for the *k*th layer [25].

(4) $$\left[T\left(U\right)\right]=\frac{1}{2}\iint^{}\left[\overset{n}{\underset{k=1}{\sum }}\int_{h_{k}}^{h_{k+1}}\rho_{k}\left\{\begin{array}{ccc} {\dot{u}}_{k} & {\dot{v}}_{k} & {\dot{w}}_{k} \end{array}\right\}{\left\{\begin{array}{ccc} {\dot{u}}_{k} & {\dot{v}}_{k} & {\dot{w}}_{k} \end{array}\right\}}^{T}dz\right]dxdy$$

The potential energy function [P(U)] because of the strain energy and transverse harmonic force is shown in Equation (5).

(5) $$\left[P\left(U\right)\right]=\frac{1}{2}\iint^{}\left[{\overset{n}{\underset{k=1}{\sum }}\int_{h_{k}}^{h_{k+1}}\left\{\sigma \right\}}^{T}\left\{\varepsilon \right\}\mathrm{dz}\right]dxdy-\int_{A}Fw_{0}dA$$

Based on the incremental matrices approach used by Rajasekaran and Murray [26] the above equation can be written as [25]:

(6) $$\left[P\left(U\right)\right]={\left\{U\right\}}^{T}\left[\left(1/2\right)K+\left(1/6\right)K_{1}\left(U\right)+\left(1/12\right)K_{2}\left(U\right)\right]\left\{U\right\}-{\left\{U\right\}}^{T}\left\{F\right\}$$

where, [*K*] stands for linear stiffness matrix, [*K***_1_**] and [*K***_2_**] stands for nonlinear stiffness matrix, which are linearly and quadratically dependent on the field variable.

For obtaining the element-level governing equations, the kinetic energy may be written as [19]:

(7) $$\left[T\left(U\right)\right]=\frac{1}{2}{\left\{\dot{\delta }\right\}}^{T}\left[M\right]\left\{\dot{U}\right\}$$

With Rayleigh proportional damping included to account for damping in the system, the governing equations of motion are as follows [19,25]:

(8) $$\left[M\right]\left\{\ddot{U}\right\}+\left[C\right]\left\{\dot{U}\right\}+\left[K+\left(1/2\right)K_{1}\left(U\right)+\left(1/3\right)K_{2}\left(U\right)\right]\left\{U\right\}=\left\{F\right\}$$

The governing Equation (8) is computed in the time domain incorporated with the shooting methodology, the Newmark time integration method, and the Newton–Raphson iteration approach, as can be shown in detail in the authors’ work [25,27], which is not included for brevity. The damping matrix [C] is calculated using the Rayleigh proportional damping model as follows [25]:

[C]=α[M]+β[K]

where $\beta =\frac{\xi }{2\omega_{n}}$; $\alpha =2\xi \omega_{n}$; (ξ represent modal damping factor; $\omega_{n}$ stands for natural fundamental frequency; and [*K*] is the linear stiffness matrix).

The present study is performed using *C*^0^ eight-noded quadrilateral shear-flexible elements having five degrees of freedom $\left(u_{0},v_{0},w_{0},\phi_{x},\phi_{y}\right)$. The field variables are expressed in terms of nodal values by using the shape functions as [25]:

(9) $$\left(u_{0},v_{0},w_{0},\phi_{x},\phi_{y}\right)=\overset{8}{\underset{i=1}{\sum }}N_{i}^{0}\left(u_{0i},v_{0i},w_{0i},\phi_{xi},\phi_{yi}\right)$$

where $N_{i}^{0}$ are the initial shape functions for the eight-noded quadratic element.

The overall response is produced in two steps: (i) the forcing frequency is raised starting at a frequency considerably away from resonance, and the convergent solution is obtained; and (ii) whenever the slope of the response curves changes abruptly or bifurcation points are encountered, the solution is proceeded with arc-length/pseudo-arc-length continuation. The eigen values of the monodromy matrix are obtained near the bifurcation points, which throw insight into the ensuing bifurcations. It is important to mention here that the shooting approach itself generates a monodromy matrix as a by-product thereby making this scheme computationally efficient; further, the banded nature of the matrices are prevented using the modified shooting method. 

## 3. Validation

The initial layer in the analysis corresponds to the bottom-most layer, and it is assumed that all layers are of same thickness. As indicated in Figure 1b, the fiber angle is selected with reference to the meridional direction. The solution procedure used in this paper is verified for the results available in the literature; the dimensionless free vibration frequencies for the clamped rectangular composite laminated plates ((0^0^/90^0^/0^0^), E_1_/ E_2_ = 40, E_2_ = 1 GPa, G_12_/E_2_ = G_13_/E_2_ = 0.6, G_23_/E_2_ = 0.5, ν_12_ = 0.25, ρ = 1000 kg/m^3^)) subjected to the transverse load are obtained and the results are presented in Table 1. The free-vibration frequencies ($\bar{\omega }$) obtained are in excellent agreement with the frequencies investigated earlier (Liew et al. [28]; Ferreira and Fasshauer [29]; Ngo-Cong et al. [30]).

## 4. Results and Discussion

The study employs 10 × 10 spatial discretization of the rectangular laminated plate with a time step of Δ*t* = π/100*ω_F_* based on the convergence steady. The layers are all the same thickness, and the first layer refers to the bottom layer. The material attributes employed in the study are as follows, unless otherwise specified:

**E**_1_/**E**_2_ = 25, **E**_2_ = **E**_3_, **G**_12_/**E**_2_ = **G**_13_/**E**_2_ = 0.5, **G**_23_/**E**_2_ = 0.2, **ν**_12_ = **ν**_23_ = **ν**_13_ = 0.25 and **E**_2_ = 1 GPa, ***ρ*** *=* 1000 kg/m^3^. All-clamped-edge (*u*_0_ *= v*_0_ *= w*_0_ *=* ϕ*_x_ =* ϕ*_y_ =* 0) boundary conditions are used in this study.

The geometrically nonlinear and linear frequency response curves are compared, demonstrating that the linear displacement/stress amplitude is much larger than predicted by the nonlinear strain–displacement relation. To explain the occurrence of higher harmonics in the overall response, the steady-state displacement/stress history and phase plane graphs have been shown. The contribution of higher harmonics to the overall response is assessed using the response’s frequency spectra. The periodic fluctuation of the steady-state fiber direction and transverse to fiber direction stress, as well as its FFT, are derived to shed light on fatigue behaviour under linear and nonlinear analysis. The nonlinear dynamic behaviour of the plate is described using stress–strain fluctuation along the thickness to indicate the amount of the tensile/compressive portion and to explain the restoring force dynamics resulting in the observed response. A systematic parametric study is used to explore the effect of fiber orientation on the nonlinear/linear steady-state forced vibration response. The geometrically nonlinear/linear (GNL/GL) peak displacement amplitude obtained based on linear and nonlinear displacement relations and percentage difference in peak amplitudes of the composite laminated rectangular plates considered in the analysis are shown in Table 2 for various fiber orientation.

**Figure 2:**
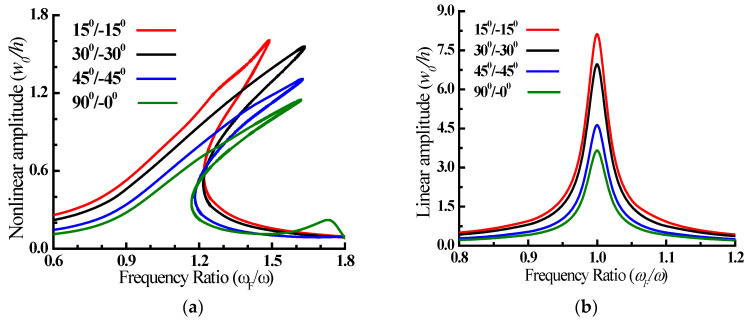
Nonlinear (**a**) and linear (**b**) variation of amplitude (*w*_0_*/h*) at the centre of the plate for different forcing frequency ratio.

The influence of the fiber angle on the nonlinear/linear forced vibration response of the all-clamped-edge, two-layered angle-ply laminated plate (*l/b* = 1, *b/h =* 100, **ζ** = 0.01, *b =* 0.5 m, *F_0_* = 100 Pa) exposed to uniformly distributed harmonic force is examined and are presented as frequency response curve (Figure 2). Table 2 presented the fundamental frequency (Hz) of a two-layered angle-ply plate which increases as the fiber angle increases. When compared to the angle-ply plate, the cross-ply plate exhibits more hardening nonlinear behaviour and a lower peak amplitude. As the fiber angle rises, the hardening nonlinearity increases and the peak displacement decreases, as seen in Figure 2. Figure 2 shows that forcing frequency ratio corresponding to the nonlinear peak displacement falls with the increasing fiber angle, with *ω_F_/ω* = 1.64, 1.558 and 1.308 for a two-layered angle-ply plate with fiber angle 15°/−15°, 30°/−30° and 45°/−45°, respectively.

Peak amplitude reduces with the increasing fiber angle in both linear and nonlinear analyses for the two-layered angle-ply plate because of greater lamination scheme-generated bending–stretching coupling, leading to higher rigidity. The comparison of peak displacement amplitude of linear and nonlinear analysis shows that linear analysis peak displacement amplitude is considerably greater than the nonlinear analysis. The comparison of linear and nonlinear peak displacement amplitude reveals that the percentage difference between the two decreases with the increase in fiber angle, and the linear peak amplitude is 5.6, 4.9 and 3.9 times the nonlinear peak amplitude for the two-layered angle-ply plate with fiber angles of 15°/−15°, 30°/−30° and 45°/−45°, respectively.

**Figure 3:**
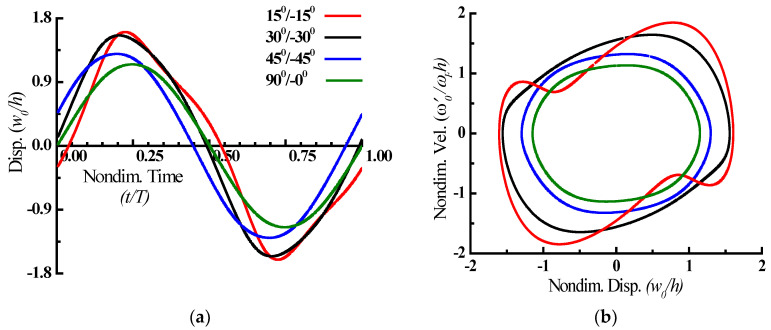
Nonlinear steady-state periodic response (**a**) and phase-plane plots (**b**) referring to the peak amplitude.

The nonlinear dynamic behaviour of the plate is evaluated by obtaining the displacement time history of the plate’s centre referring to the forcing frequency ratio of peak amplitude in Figure 2’s nonlinear response, which is shown in Figure 3 together with the phase-plane plot at this moment. The steady-state response history shows that the positive/negative half-cycle amplitudes will have nearly equal intervals in tension and compression for all fiber angles evaluated. The asymmetricity in the phase-plane plot implies strong higher harmonic participation, whereas the symmetric phase-plane plots imply limited higher harmonic involvement.

**Figure 4:**
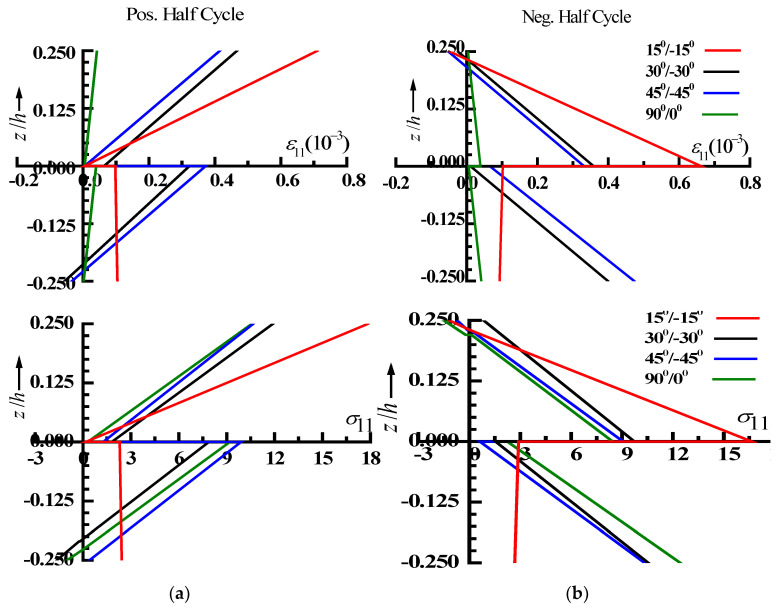
Nonlinear fiber direction normal strain and stress (ε_11_ & σ_11_) distribution for positive half-cycle (**a**) and negative half-cycle (**b**).

Figure 4 depicts the variation of the nonlinear steady-state normal strain/stress throughout the thickness of laminate in the positive/negative half-cycle at forcing frequency ratio of the peak amplitude. Figure 4 shows that the top layer of the plate is in the tension for all fiber angles considered in the case of positive half-cycle, whereas the bottom layer is in tension for the negative half-cycle.

**Figure 5:**
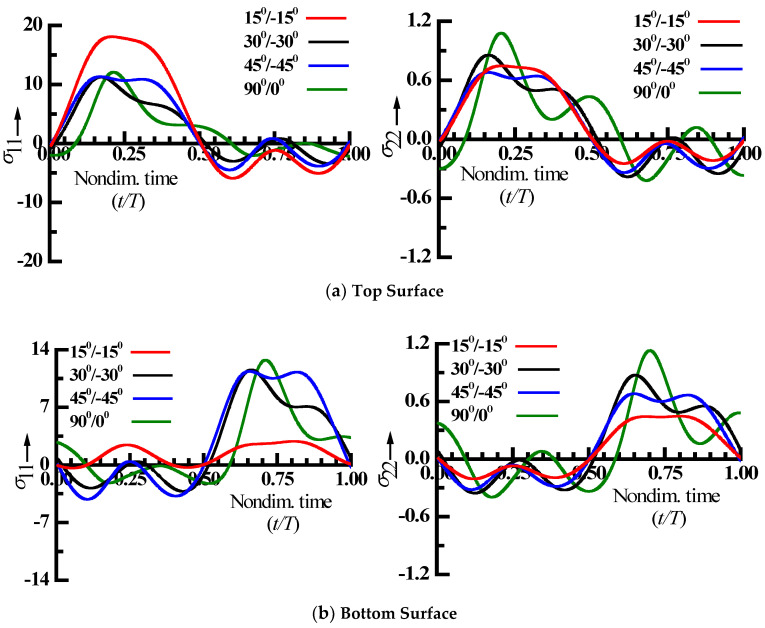
Nonlinear periodic stress (σ_11_ and σ_22_) distributions for top (**a**) and bottom surface (**b**), respectively.

The fluctuation of the nonlinear steady-state normal stress (Fiber-direction “σ_11_” and transverse to fiber direction “σ_22_”) within a time period at the centre of the top/bottom surface with reference to forcing frequency ratio of the peak displacement amplitude in the nonlinear response is presented in Figure 5. It is observed from Figure 5 that for the two-layered plate with all the fiber angles considered, the tensile stress amplitude (σ_11_ and σ_22_) at the top/bottom surfaces are greater than compressive stress amplitude. The positive and negative half-cycle times of both top/bottom surfaces are nearly equal for all the fiber orientations, except for the 15°/−15° angle-ply laminated composite plate. The cyclic stress variation reveals multiple stress reversals and slope changes within the loading cycle for all the load, revealing higher harmonic contribution, which is critical for the laminated composite plate’s fatigue design [27].

**Figure 6:**
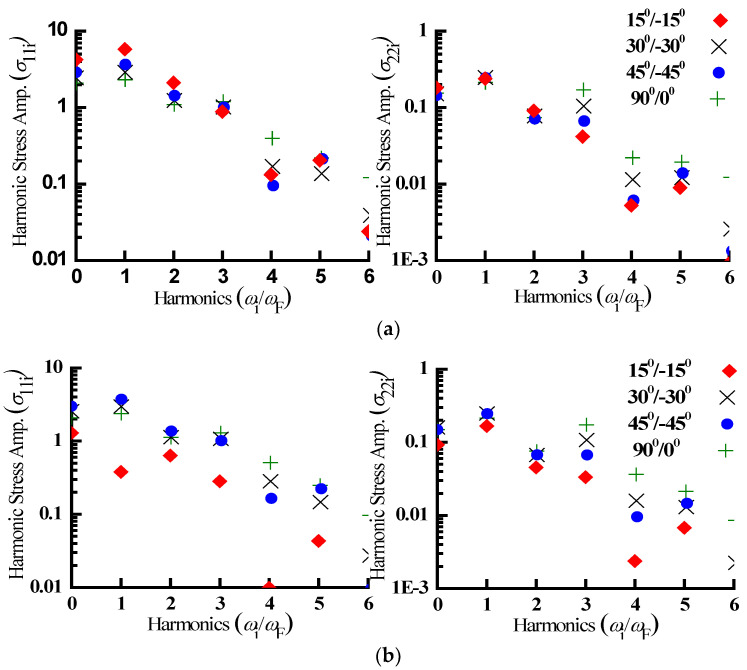
FFT of the steady-state stress referring to Figure 2 for top surface (**a**) and bottom surface (**b**), respectively.

The contributions from the higher harmonics as revealed from the cyclic variations of the nonlinear stress were quantified by obtaining the frequency spectrum of nonlinear steady-state stresses (σ_11_ and σ_22_) employing the fast Fourier transform (FFT), and the findings are shown in the Figure 6. FFT shows a significantly greater higher harmonics participation together with the fundamental harmonic for all fiber orientations. The second harmonic contributions for two-layered angle ply plate with 15°/−15° lamination scheme at the centre of the bottom surface is greater than the fundamental harmonic. This is because quadratic nonlinear restoring forces are stronger than quadratic nonlinear restoring forces as well as linear restoring forces [27].

## 5. Conclusions

The steady-state nonlinear/linear periodic response studies of a laminated composite rectangular plate for different fiber orientations were analyzed. To demonstrate the variation of nondimensional peak displacement amplitude with the forcing frequency ratio, the linear/nonlinear frequency response curves were plotted. The steady-state response displacement/stress history, phase-plane plots, and the FFT of response were used to investigate the nonlinear dynamic behaviour. The response’s peculiarity is explained by the fluctuation of strain/stress over the laminate’s thickness. The following are the main observations drawn:

- When compared to the angle-ply plate, the cross-ply plate exhibits more hardening nonlinear behaviour and a lower peak amplitude. As the fiber angle rises, the hardening nonlinearity increases and the peak amplitude drops.
- Variations in nonlinear stresses throughout a loading cycle indicate repeated slope changes and stress reversals, suggesting the presence of fluctuating stresses, which is crucial for fatigue design.
- The frequency spectra of nonlinear steady stress displays significant higher harmonic contributions, and in some circumstances, second/third harmonic contributions are greater/comparable to fundamental harmonic contributions. Greater even-order harmonics result from a higher contribution of quadratic nonlinear restoring forces, whereas higher odd-order harmonics result from a greater participation of cubic nonlinear restoring forces.

## Figures and Tables

**Table 1:** Free-vibration dimensionless frequencies ($\bar{\omega }=\left(\frac{b^{2}}{{}^{2}}\right)\sqrt{\frac{\rho h}{D_{0}}})\;$.

*a/b*	*t/b*	Study	Mode Sequence Number			
			1	2	3	4
**1**	**0.001**	**Present**	**14.6736**	**17.6454**	**24.6955**	**36.2302**
		Ngo-Cong et al. [30]	14.6722	17.6383	24.5238	35.4471
		Ferreira and Fasshauer [29]	14.8138	17.6138	24.5114	35.5318
		Liew et al. [28]	14.6655	17.6138	24.5114	35.5318
	**0.2**	**Present**	**4.4587**	**6.6623**	**7.7246**	**9.2185**
		Ngo-Cong et al. [30]	4.4466	6.6419	7.6996	9.1852
		Ferreira and Fasshauer [29]	4.4463	6.6419	7.6995	9.1839
		Liew et al. [28]	4.4468	6.6419	7.6996	9.1852
**2**	**0.001**	**Present**	**5.1079**	**10.5547**	**10.6112**	**14.4045**
		Ngo-Cong et al. [30]	5.1092	10.5447	10.6042	14.3642
		Liew et al. [28]	5.1051	10.5265	10.5828	14.3241
	**0.2**	**Present**	**3.0516**	**4.2603**	**5.8075**	**5.9263**
		Ngo-Cong et al. [30]	3.0453	4.2484	5.7917	5.9050
		Liew et al. [28]	3.0453	4.2484	5.7918	5.9047

**Table 2:** Fundamental free-vibration frequencies (Hz) and peak amplitudes.

Load (Pa)	*a/b*	Lamination Scheme	Fundamental Frequency (Hz)	Nondimensional Amplitude (*w*_0_*/h*)		% Difference of GNL/GL Amplitudes
				GNL	GL	
100	1.0	15°/−15°	31.645	1.606	9.000	460.39
		30°/−30°	34.012	1.558	7.715	395.19
		45°/−45°	41.584	1.308	5.129	292.13
		90°/0°	47.280	1.149	4.050	252.48

## Data Availability

Not applicable.
